# Risk factors for severe COVID-19 illness in healthcare workers: Too many unknowns

**DOI:** 10.1017/ice.2020.178

**Published:** 2020-04-27

**Authors:** Pandora L. Wander, Marika Orlov, Susan E. Merel, Daniel A. Enquobahrie

**Affiliations:** 1 Division of General Internal Medicine, Department of Medicine, University of Washington, Seattle, Washington; 2 Department of Veterans Affairs, VA Puget Sound Health Care System, Seattle, Washington; 3 Department of Epidemiology, University of Washington, Seattle, Washington

*To the Editor*—We were very interested to read the recent letter by Zhou et al, “Protecting Chinese Healthcare Workers While Combating the 2019 Novel Coronavirus.”^1^ We agree that everything possible should be done to protect healthcare workers (HCWs) from developing COVID-19. We agree with the recommendations of Zhou et al including the importance of increasing production of personal protection equipment (PPE), training HCWs in proper use, and maintaining a high clinical suspicion for COVID-19 even in patients without respiratory symptoms. In addition, we call for more research into the risk factors leading to severe illness among HCWs, defined as COVID-19 requiring hospitalization or admission to the intensive care unit.^2^ Research in this area is sorely lacking, limiting implementation of evidence-based practices. Despite being younger and healthier than the general population with COVID-19, HCWs have similar rates of severe illness. In China, the proportion of HCWs with severe illness decreased from 45% in early January 2020 to 9% after February 1,^3^ likely reflecting more consistent adoption of appropriate infection-control practices, including the use of PPE. However, severe illness among HCWs continues to be reported, suggesting that the use of currently approved infection control processes do not entirely prevent severe COVID-19 among HCWs. To explore risk factors for severe COVID-19 in HCWs, we performed structured searches using a Twitter analytics tool (Tweet Archivist, Seattle, WA) to identify news stories reported before March 16, 2020, about HCWs with severe COVID-19–related illness (n = 6 cases) (Table 1). In most of these articles, neither high-risk host factors nor a clear high-inoculum exposure was evident, but in a few reports, potential exposure to inocula containing a high viral load was reported, including potential exposures to virus in stool.

**Table 1.** Healthcare Worker Cases of Severe COVID-19 Reported in the Media Before March 16, 2020

Case No.	Age, y	Sex	Occupation	Location	Host Factors	Exposure Factors	Ref	Hypothesized Mechanism of Large-Inoculum Exposure
1	29	F	Gastroenterologist	Wuhan, China	None known	Frequent bedside visits to 76 year-old man with suspected COVID-19, had symptoms 5 days later; “took precautions” to protect herself	8	Virus in stool; whether colonoscopy was done was not reported
2	29	F	Nurse	Wuhan, China	None known	Visited families of confirmed cases, teaching how to disinfect their homes; “took precautions” to protect herself	8	Unknown, potentially could have been exposed to large inoculum cleaning stool or other waste in homes of confirmed cases
3	34	M	Ophthalmologist	Wuhan, China	None known	Treated a patient with acute angle-closure glaucoma	9	Sustained, close contact during angle-closure glaucoma procedure
4	40-49	M	ED physician	Washington	None known	“Complied at all times with appropriate PPE procedures”	10, 11	Unknown
5	70	M	ED physician	Paterson, New Jersey	Age	“Leads his institution’s emergency preparedness”	11	Unknown
6	67	M	General practitioner	Varese, Italy	Age	Quoted as saying, “We have run out of masks, but we don’t stop. We are careful, and we go on.”	12	Continued working without PPE

Note. ED, emergency department; PPE, personal protection equipment.

In 10% of cases, gastrointestinal symptoms precede fever or respiratory symptoms by 1–2 days.^4^ Furthermore, 60% of samples from the toilet, sink and door handles of an individual with SARS-CoV-2 were positive for viral RNA, even though the individual reported respiratory symptoms but not diarrhea.^5^ For HCWs, contact with surfaces and/or patients with these symptoms could represent opportunities for high-inoculum exposure. During the SARS epidemic, SARS-CoV RNA was detected in stool in greater quantities than any other site,^6^ leading the World Health Organization to conclude that, “diarrhoea could still remain important for infectivity, regardless of its cause.” Although SARS-CoV-2 RNA is readily found in stool,^7^ whether replicating virus is present is less clear. However, ACE2 receptors, which are used by the virus to infect cells, are present in the GI tract,^7^ making it plausible that the GI tract is an active site of viral replication. We therefore postulate that exposure to virus from high-viral load sites such as stool should be formally evaluated as an ongoing risk factor for severe COVID-19–related illness in HCWs. To facilitate research in this area and to ensure adequate power, we suggest that deidentified information about HCW cases be shared in national data repositories so that these and other risk factors can be assessed and the workforce can be adequately protected. In the meantime, institutions, if not already doing so, should screen for diarrheal symptoms.
